# Secure E-Health Platform

**DOI:** 10.1007/978-3-030-51517-1_20

**Published:** 2020-05-31

**Authors:** Karima Djouadi, Abdelkader Belkhir

**Affiliations:** 8 Digital Research Centre of Sfax, Sfax, Tunisia; 9 Institut Mines-Télécom, CNRS, Paris, France; 10 Université de Sherbrooke, Sherbrooke, QC Canada; 11 Digital Research Centre of Sfax, Sfax, Tunisia; 12 University of Sfax, Sfax, Tunisia; Department of Computer Science, Computer Systems Laboratory, USTHB BO 32, Bab Ezzouar, 16111 Algiers Algeria

**Keywords:** Internet of things (IoT), E-health, Security, Confidentiality

## Abstract

Currently, the Internet has become a service hosting infrastructure through its interconnection of a very large number of heterogeneous objects, thus offering users several types of services implemented by different sectors. Although these services make people’s lives easier and provide them with a means of communication between their real and virtual worlds, they risk being a path of intrusion into their private lives, or in some cases an easy target for malicious individuals aiming to endanger human life. To avoid this, we have designed a secure e-health platform based on IoT that serves to monitor patients’ medical profiles remotely by collecting their medical records while ensuring their confidentiality and integrity.

## Introduction

With the interconnection of billions of objects around the world, IoT offers several services to individuals through many types of applications deployed in several domains, including smart grid [1], smart home, smart city, smart healthcare and applications dedicated to vehicle monitoring [2, 3].

Healthcare Systems have emerged to address some of the problems facing the health sector, mainly the lack of medical staff caused by the ever-increasing population and the lack of timely diagnosis of diseases [4] by allowing constant medical monitoring of chronic patients or residents of isolated or underserved locations. In this development and deployment of e-health systems, information management by mobile devices require very short response times and latency, it introduces also several challenges including data storage and management, security and confidentiality (e.g. authorization control and anonymity) [8]. The integration of fairly strong security mechanisms is necessary for this systems where a successful security attack results in several human lives being subjected to false diagnosis or delayed surgical procedures.

We are interested to problems of storage, confidentiality and data integrity by ensuring minimal response time and low latency. Several authors have conducted works aimed at setting up remote patient monitoring platforms such as [13–16]. Nevertheless, this works present some limitations such as neglecting the security of the monitoring systems, the privacy of its users, the availability and the storage of data.

To address the aforementioned issues, we propose a solution to provide the healthcare community and patients with a secure medical service. Our platform offers continuous medical monitoring with the integration of medical data backup mechanisms at the Cloud level, thus ensuring the notion of fault tolerance through data replication and thus the availability of information while guaranteeing the integrity and confidentiality of the data exchanged ( using sh1 and MD5 protocols for hashage and DES ,RSA for encryption), a minimal response time and low latency due to the implementation of fog computing. The remainder of this paper is organized as follows. Section 2 reviews related works on healthcare systems. Section 3 describes the proposed solution. Section 4 presents the experiments. Finally, Sect. 5 concludes the paper.

## Related Works

In this section, we discuss the related work of healthcare systems. In [5], The authors present a cloud computing solution for patient’s data collection in healthcare institutions. The system uses sensors attached to medical equipment to collect patient data and sends it to cloud for providing ubiquitous access. In [6], the proposed architecture is dedicated to data acquisition via several personal health devices via USB, ZigBee or Bluetooth. But the disadvantage of the above-mentioned work is that the response time and latency increases due to the long path to the cloud, which influences the user’s access time to the data. In [7] who have implemented an IoT- healthcare system architecture which benefits from the concept of fog computing, thus ensuring low latency data processing and low bandwidth usage. The main disadvantage of the above works is the neglect of the notion of security. In [15] authors have proposed a robust solution in terms of response time by ensuring the confidentiality of data via an authentication protocol except that it only authenticate LPU (Local Process Unit) and not identify the users and neglects the property of data availability by centralizing storage at the level of a single server, in the event of failure of the latter, access to medical records will be suspended, which could endanger human life. While the authors of [16] Propose a secure healthcare system with the same drawback as the previous one with neglect of the quality of service criteria (response time and latency). On the other hand, our approach maintains a backup procedure to ensure continuity of service while guaranteeing the unique identity rule via the NIN [12]. According to [17] that uses Blockchain as a security method offers several advantages by allowing an agreement without the use of a trusted third party and thus avoiding the bottleneck, the antecedent medical data are also complete and coherent thanks to the chaining. However, this technology requires a significant investment which is very costly. It should also be noted that the blockchain consumes a lot of computing time, which is not ideal for e-health platforms.

## E-Health Platform

In this section we present the architecture of our IoT system which is based on fog-enabled cloud computing as described in [10]. Then we will present the two main processes of our system and we finish by presenting the different security aspects available on our platform.

### System Architecture

**Fig. 1.**
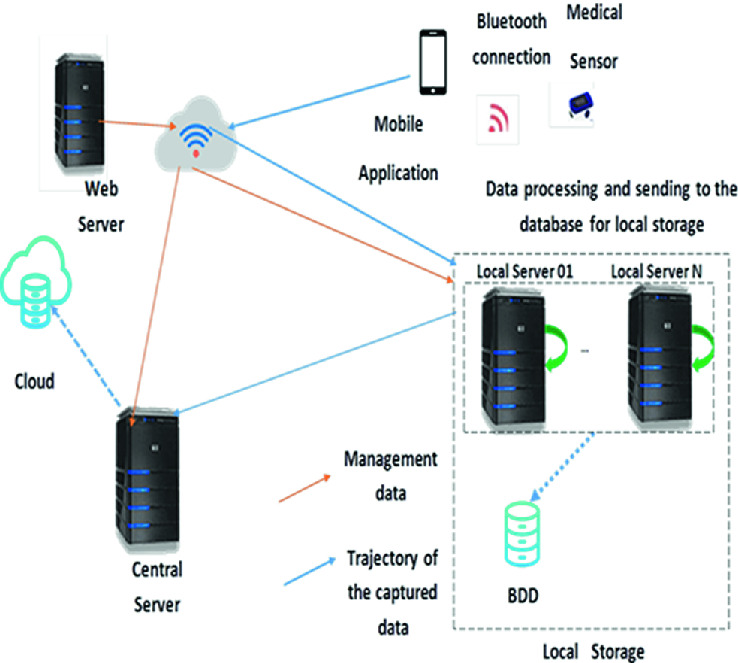
E-health platform

As illustrated in Fig. 1, the architecture of our system is mainly based on (N) local servers distributed geographically over (N) zones, in which patients and medical institutions can be located, medical sensors used for the collection of patients data and IoT equipment (smartphones); as well as a central server (cloud) dealing with global data storage.

The different actors and components of our architecture are explained as follows:

- **Medical sensors:** it is planted in, on or around a human body to capture the patient’s health data and send it to the system periodically.
- **User:** consists of two categories of users, each of them has a unique identifier (NIN) [12]:
  1. *The Patient*: person with whom one or more sensors are associated to monitor its health status.
  2. *The practitioner*: person who is in charge of setting up the association between the patient and his sensor.
- **Mobile application:** a User Interface (UI); it authenticates users by submitting their identifiers (NIN) [12] with their relative passwords. It also allow the Patient/sensor association procedure, which only the practitioner is authorized to do, it allows him to consult the medical records of his patients and make diagnoses.
- **Local servers:** a distributed computing paradigm that acts as an intermediate between Cloud datacenters and IoT sensors. Its role is to lightly process the medical data collected by the sensors associated with the different patients geographically distributed over the (N) zones, followed by the backup of this data on local databases.
- **Web server:** administrates and maintains the system, including the management of the accounts of the administrator of the (N) area. All this is done by the super administrator, while each administrator is responsible for creating and managing the accounts of the users in his or her zone.
- **Cloud:** a central server, which is responsible for the global storage of medical and personal data of users in all areas, with the possibility of transferring this data to an area X to which the patient has moved, if the attending physician wants to consult the file of his new patient, who has just arrived in his new area.

### The Main Operating Processes

Among the most important processes for the realization of our IoT service platform, the following two processes driving the operating principle of the mobile application, the association of sensors to patients and sends it as well as data storage.

- **Process of association’s creation**
  The practitioner first searches the patient’s profile at the local level by introducing his identifier, otherwise, the local server sends a request to the central server of the Cloud to retrieve and save it at its level.
  After retrieving the patient’s profile, practitioner checks the availability of the sensor to associate it with the right patient; This procedure can be summarized by the following steps:
  1. Initiation of the association process by the practitioner.
  2. Sensor search procedure and availability test.
  3. Beginning of capture of the patient’s medical constants.
  In the case of healing of the patient or the end of his or her followup by his practitioner, the latter ends this association by disassociating the patient’s sensor.
- **Process of detection and data transmission by sensors**
  After the patient sensor association is established, the sensor begins to capture the patient’s data and sends it through the mobile application to the local server for storage.

### Security of the E-Health Platform

We introduced security mechanisms to protect the captured user data as it will be sent to processing equipment and then to storage spaces, which will make them subject to theft or alteration attempts [3]which represent very high risks for the functioning of IoT applications.

- **Data integrity: ** Data integrity refers to the state of data that, at the time of processing, storage or transmission is not intentionally or accidentally altered or destroyed and maintains a format that allows its use. For this purpose, the programmed SQL queries will be used, and data will be encrypted using the RSA asymmetric encryption algorithm, to ensure such data integrity.
- **Backup and logging of the cloud database: ** To preserve the data within our database, we have opted for the backup strategy which consists of making copies of existing data in order to improve reliability, fault tolerance, or availability. Every day, our database will be automatically replicated to the cloud to ensure continuity of service in the event of a local server failure.
- **Authentication: ** The authentication process will allow us to prevent privacy breaches, unauthorized access to data, identity theft, and password attacks by limiting authentication and login attempts to the private areas of practitioner and patient users. This is done by using small gadgets called “smart cards”, which each user has. These cards have the above-mentioned user ID (NIN) and a corresponding password [12].
- **Data encryption: ** To prevent human attacks from the middle, the following encryption mechanisms are applied to the exchanged data in our system.
  - Encryption of data within the database, using md5 and DES encryption protocols.
  - Encryption of data circulating in the network, using SSL Sockets with certificates and Data hash by applying the SH1 protocol [9].

## Implementation and Results

In this section we present the basic implementation setup, then we introduce the mobile prototype to validate our solution as well as some simulations and discussion of results.

The experiments were conducted on an IoT sensor Xiaomi Mi Band3 [11] is an intelligent IoT-based electronic bracelet that incorporates an HR (Heart Rate) heart rate sensor. The user interface (UI) was built using an Android Studio V4.4+. We have used the Google Cloud Platform for the creation of our local servers and the global server for the data storage.

### Mobile Application Prototype

**Fig. 2.**
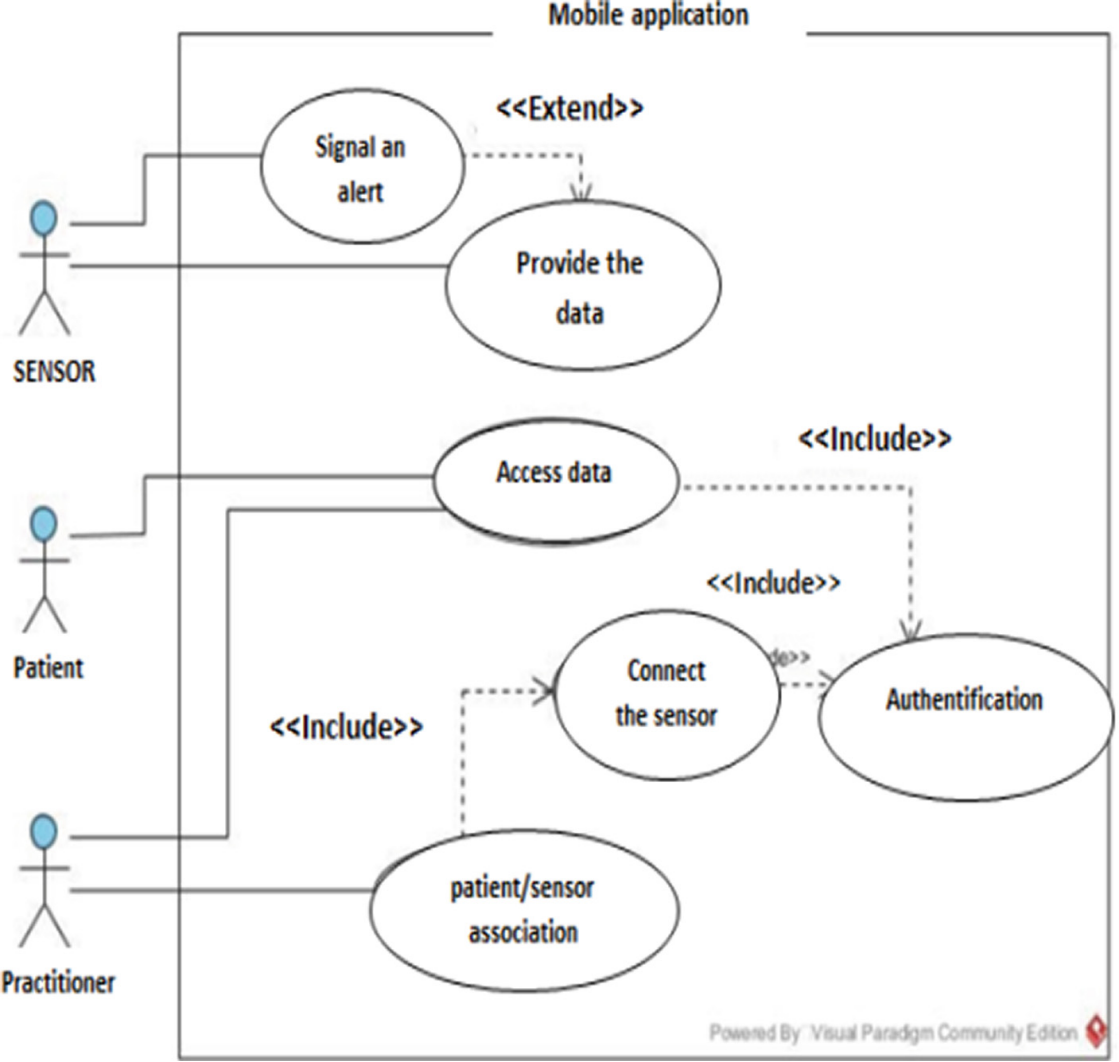
Use case of the mobile application

The mobile application is used to communicate with the IoT device in order to collect the data. It will be installed at the practitioner and the patient having a profile for each one of them (see Fig. 2). Both types of users will be entitled to the following functionalities:

- **For the practitioner:** Patient/Sensor Association, Monitoring,Transfer, and patients update.
- **For the patient:** consultation of his various medical information.

As can be seen in Fig. 2, each time the sensor registers a new value; it sends it to the mobile application to which it is connected. In the case of a heart defect as shown in the following figure, notifications and alerts are sent to the practitioners treating the patient, and a telephone call is made from the patient’s home to the toll-free civil protection number.

### Simulation and Discussion of Results

In order to test the performance of our system, and see how it will react to changes in the number of users, simultaneous access to the server through the use of “Threads” and the data encryption operation; we launched a simulation with a series of tests based on the response time of the local server for requests issued by the users, whether patients or practitioners, as this time may be a factor in the favorable to saving a human life.

**Test 1:** Incrementing the number of users with the use of Threads and encryption: First, we start by testing the incrementation of the number of users with the activation of simultaneous access to the server using “Threads”, and the encryption and data encryption operation.

**Test 2:** Incrementing the number of users with the use of Threads without encryption: Secondly, we test the incrementation of the number of users with the activation of simultaneous access to the server using “Threads”, and by disabling the encryption and data encryption operation.

**Test 3:** Incrementing the number of users without Threads and encryption: Finally, we tested the incrementation of the number of users without the activation of simultaneous access by using “Threads”, and without encryption.

**Fig. 3.**
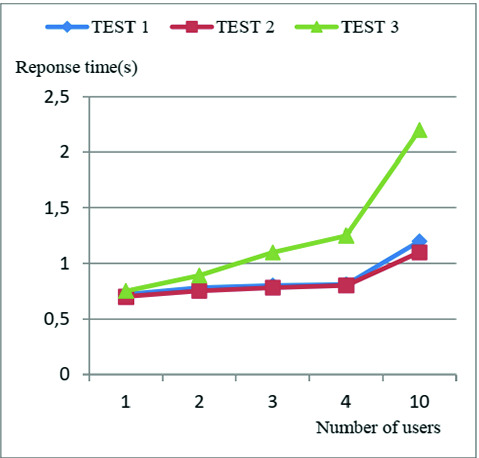
Simulation’s tests

After completing the series of tests, and as illustrated in Fig. 3 We noticed that the encryption operation will not have much influence on the system’s response time. Whilst, the use of Threads will have an impact on the load on the communication network. After analyzing the simulation results, we conclude that as the number of users increases, the number of requests to be processed by the server increases too, which will lead to congestion at the server level and additional traffic on the network, thus prolonging the response time, which is not tolerable in our system in the event of an extreme emergency. Whilst with the use of Threads, We notice that the different requests will be processed simultaneously, which will, therefore, reduce the response time, as well as the load on the network.

## Conclusion and Perspectives

We have developed a service platform based on IoT, with a secure online health application to establish the medical profiles of patients from distributed information, allowing their continuous monitoring in order to improve and modernize health services by ensuring the security of the data exchanged on our platform, thus guaranteeing the preservation of patients’ privacy.As a future work, We plan to expand the real deployment of integrated sensors to better evaluate our system.
